# Stellenwert der notfallmäßigen temporären Fixateur-externe-Anlage bei bikondylären Tibiakopffrakturen: Eine 10-jährige retrospektive Analyse eines universitären Level-I-Traumazentrums

**DOI:** 10.1007/s00113-025-01604-8

**Published:** 2025-07-25

**Authors:** Claas Neidlein, Daniel P. Berthold, Felix Winden, Boris Michael Holzapfel, Wolfgang Böcker, Julian Fürmetz, Markus Bormann

**Affiliations:** 1 Muskuloskelettales Universitätszentrum München, Ludwig-Maximilians-Universität München (LMU), Marchioninistraße 15, 81377 München, Deutschland; 2 Klinik für Arthroskopische Chirurgie, Sporttraumatologie und Sportmedizin, BG Klinikum Duisburg, Universität Duisburg-Essen, 47249 Duisburg, Deutschland; 3 Sporttraumatologie und Arthroskopische Chirurgie, BG-Unfallklinik Murnau, Professor-Küntscher-Straße 8, 82418 Murnau, Deutschland

**Keywords:** Tibiakopffraktur, Bikondyläre Tibiakopffraktur, Komplikationen, Fixateur externe, Osteosynthese, Tibial plateau fracture, Bicondylar tibial plateau fracture, Complications, External fixation, Osteosynthesis

## Abstract

**Hintergrund:**

Tibiakopffrakturen (TKF) verzeichnen in den letzten 10 Jahren einen signifikanten Inzidenzanstieg. Häufig handelt es sich hierbei um bikondyläre Frakturen. Die initiale Versorgung dieser Frakturen mittels Fixateur externe (tEF) wird kontrovers diskutiert, und es gibt nur wenige Daten über Komplikationen bei temporär angelegtem tEF.

**Ziel der Arbeit:**

Das Ziel dieser Studie ist, die Komplikationsraten zwischen Patienten mit und ohne initiale tEF-Anlage in einem ähnlichen Patientenkollektiv zu untersuchen.

**Material und Methoden:**

Diese monozentrische retrospektive Studie analysiert die Komplikationsraten von bikondylären Tibiakopffrakturen von Januar 2011 bis Dezember 2020 an einem universitären überregionalen Traumazentrum. Die Frakturen wurden nach der jeweiligen initialen Therapie in 2 Gruppen eingeteilt (temporärer Fixateur externe (tEF)) und primäre Immobilisierung in Gips/Orthese (iGO). Das Komplikationsrisiko wurde mithilfe einer univariaten Regressionsanalyse ermittelt.

**Ergebnisse:**

Insgesamt zeigte sich kein signifikant erhöhtes relatives Risiko für Komplikationen zwischen tEF und iGO (Odds Ratio (OR) 1,97, 95 %-KI 0,90–4,37, *p* = 0,069). Die spezifische Komplikation einer postinterventionellen Infektion trat jedoch signifikant häufiger bei der Verwendung von tEF auf (OR 5,12, 95 %-KI 1,27–29,88, *p* = 0,01), während die Gesamtrate der Komplikationen keine signifikante Zunahme zeigte. Der Einsatz von tEF war nicht mit einem eingeschränkten Bewegungsmaß (ROM) assoziiert.

**Diskussion:**

Die postoperative Gesamtkomplikationsrate bei Tibiakopffrakturen wird durch die initiale Versorgung mit einem Fixateur externe oder einem Gips nicht signifikant beeinflusst. Die Entscheidung für einen temporären Fixateur sollte individuell und basierend auf klaren Indikationen getroffen werden. Die höhere Infektionsrate bei Patienten mit einem Fixateur externe stellt ein Risiko dar; dieses muss im Zusammenhang mit einem möglichen Bias durch komplexere Weichteil- und Knochenverletzungen sowie Mehrfachverletzungen betrachtet werden.

**Graphic abstract:**

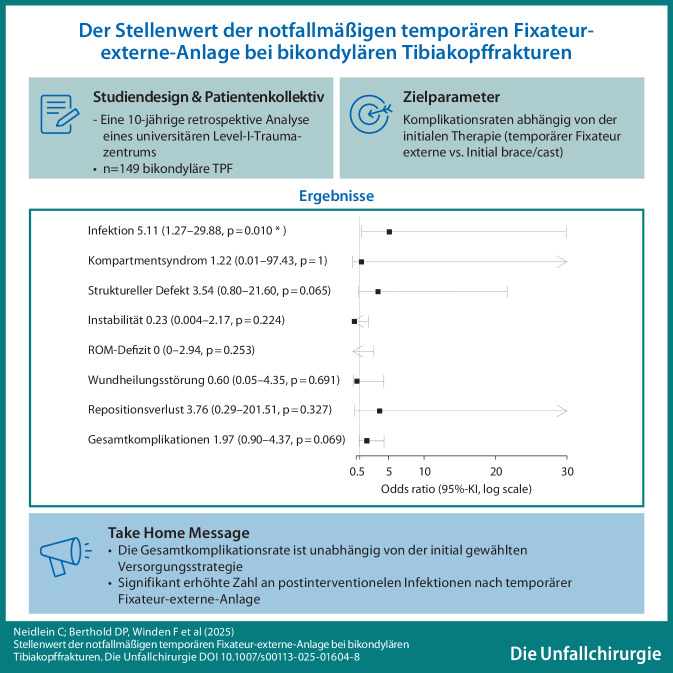

## Abstract (en)

**Background:**

Tibial plateau fractures (TPF) have significantly increased over the last decade, with a notable proportion being bicondylar fractures. The necessity of a temporary external fixator (tEF) as an initial treatment for bicondylar TPF remains controversial, with limited data available regarding associated complications.

**Aim of the study:**

The aim of this study is to investigate the complication rates between patients with and without initial treatment using tEF in patients with bicondylar TPF.

**Material and methods:**

This monocentric retrospective study analyzed the complication rates of bicondylar TPF from January 2011 to December 2020 at a university national trauma center. The bicondylar TPFs were divided into two groups based on the respective initial treatment: temporary external fixator (tEF, *n* = 67) and primary immobilization in brace/cast (iBC, *n* = 82). The complication rate was determined using univariate regression analysis.

**Results:**

Overall, there was no significantly increased relative risk of complications between tEF and iBC (odds ratio, OR 1.97, 95% confidence interval, CI 0.90–4.37, *p* = 0.069). The specific complication of a postinterventional infection, however, was significantly more frequent with the use of tEF (OR 5.11, 95% CI 1.27–29.88, *p* = 0.01) but the use of tEF was not associated with an impaired range of motion (ROM).

**Discussion:**

The overall postoperative complication rate for tibial plateau fractures is not influenced by the initial treatment with a temporary external fixator or a brace/cast. The decision for a tEF should be made individually and based on clear indications. The higher infection rate in patients with tEF represents a risk that must be considered in the context of potential bias related to more complex soft tissue and bone injuries as well as multiple injuries. Further studies are needed to validate these findings and provide further analysis to improve clinical decision making.

## Einleitung

Tibiakopffrakturen (TKF) zeigen in den letzten Jahren einen signifikanten Inzidenzanstieg [2, 6, 30]. Komplexe, bikondyläre Typ-V- und Typ-VI-Frakturen gemäß der Schatzker-Klassifikation resp. AO/OTA-41-C-Frakturen haben einen Anteil von knapp über 30 % aller TKF [2] und benötigen in der Regel eine operative Versorgung [1, 9, 13–15, 19, 29]. Die in der Literatur beschriebenen hohen Komplikationsraten (bis zu 100 %) und Raten an posttraumatischen Arthrosen (bis zu 44 %) spiegeln die chirurgische Herausforderung der operativen Versorgung wider [5, 16, 20, 25, 32, 34]. Die Versorgung wird deshalb immer häufiger von einem spezialisierten Team in Zentren durchgeführt [3, 16].

Die primäre notfallmäßige Osteosynthese dieser Frakturen stellt eine Ausnahme dar. Die operative Ausversorgung erfolgt regelhaft frühelektiv unter Verwendung geeigneter personeller und apparativer Ressourcen [4, 22]. In der Literatur wird für die primäre Versorgung ein durchschnittliches Versorgungsintervall von 4 bis 7 Tagen angegeben [10, 14, 35].

Im Fall von Luxationsstellungen, offenen Frakturen, kritischen Weichteilen und/oder sensomotorischem/neurologischen Defiziten ist eine notfallmäßige Versorgung indiziert; diese beinhaltet meist die temporäre-Anlage eines Fixateur externe (tEF; [3, 4, 29]). Eine standardmäßige Anlage von tEF bei bikondylären Frakturen wird jedoch kritisch diskutiert [19].

Eine evidenzbasierte Empfehlung zur-Anlage von tEF bei TKF fehlt, und die Indikation beruht häufig auf internen Klinikstandards oder chirurgischen Fähigkeiten.

Als Vorteile der tEF-Anlage werden die frühe Stabilisierung der Fraktur, eine Schonung der Weichteile durch kleine Inzisionen und die Verhinderung eines Gipses/Schiene genannt [3]. Einige Autoren beschreiben kein erhöhtes Infektionsrisiko durch die Anlage eines tEF [3, 16]. Andere Studien beschreiben jedoch erhöhte Infektionsraten von 2,07- bis zu 3,9fach [7, 24, 27]. Weiterhin kann durch die eingebrachten *Schanz-Schrauben* der Zugangsweg für die sekundäre osteosynthetische Versorgung eingeschränkt werden [3]. Zu den chirurgischen Risiken der tEF gesellen sich die anästhesiologischen und allgemeinen perioperativen Risiken einer notfallmäßigen Operation [33]. Jedoch bleibt bisher unklar, ob Patienten mit einer tEF-Anlage generell hinsichtlich des Outcome und begleitender Komplikationen von dieser Prozedur profitieren.

Ziel dieser Studie ist es, die frühen Komplikationsraten zwischen Patienten mit und ohne Fixateur externe in einem ähnlichen Patientenkollektiv retrospektiv über die letzten 10 Jahre an einem universitären Level-I-Traumazentrum zu untersuchen. Die Hypothese der Autoren lautete, dass die tEF-Anlage bei komplexen TKF nicht zu signifikant höheren postoperativen Komplikationsraten führt.

## Methoden

### Datenanalyse

Diese retrospektive monozentrische Studie wurde an einem universitären überregionalen Traumazentrum der Maximalversorgung durchgeführt. Die Durchführung dieser Studie erhielt die Genehmigung der lokalen Ethikkommission (21-0559) und erfolgte gemäß den Prinzipien der Deklaration von Helsinki.

Im Zeitraum vom Januar 2011 bis Dezember 2020 erfolgte eine retrospektive Datenerhebung anhand eines klinikinternen Tibiakopfregisters. Der Mindestnachuntersuchungszeitraum betrug 2 Jahre.

Eingeschlossen wurden sämtliche Patienten mit bikondylärer TKF mit intraartikulärer Beteiligung. Ausgeschlossen wurden Patienten < 18 Jahre sowie unvollständig dokumentierte Patienten (Alter, Geschlecht, Unfallzeitpunkt, Computertomographie(CT)-Bildgebung, Operationsbericht, postoperative „range of motion“ (ROM), Body-Mass-Index (BMI) und Schnitt-Naht-Zeit). Zusätzlich musste die initiale Dokumentation der peripheren Durchblutung, Motorik und Sensibilität (pDMS) vorliegen sowie die Dokumentation des initialen Weichteilstatus erfolgt sein. Der Operationsbericht der tEF-Anlage wurde retrospektiv hinsichtlich der Indikation zur tEF-Anlage ausgewertet. Die initiale Therapieentscheidung erfolgte durch den diensthabenden traumatologischen Facharzt.

### Klassifikation

Die Klassifikation der Frakturen erfolgte anhand der in der Literatur nach wie vor am häufigsten verwendeten Klassifikation nach Schatzker [31]. Auf eine zusätzliche Klassifikation wurde verzichtet, um die Übersichtlichkeit zu wahren und eine potenzielle Redundanz durch parallele Klassifikationssysteme zu vermeiden. Bei Unklarheiten und Meinungsverschiedenheiten zwischen den Untersuchern (C. N., M. B., J. F.) wurde durch gemeinschaftliche Diskussion eine Lösung gefunden.

### Komplikationen

Eine Komplikation wurde als solche gewertet, wenn es sich um eine nichtgeplante, operationsbedürftige Komplikation wie z. B. Kompartmentspaltung, oberflächliche und/oder tiefe Wundinfektion, Nerven- und Gefäßschaden oder Reosteosynthese handelte.

Erfolgte eine zweizeitige ligamentäre Operation aufgrund einer subjektiven und/oder objektivierbaren Instabilität, wurde dieser Eingriff unabhängig von der zugrunde liegenden Ursache der Instabilität (postoperative Deformität, initial nichtdiagnostizierte/-versorgte ligamentäre Verletzung etc.) als Revision gewertet.

## Statistische Auswertung

Die durchgeführte Post-hoc-Poweranalyse ergab eine Power von 99,9 %. Die statistische und grafische Analyse erfolgte mit RStudio (Firma Posit PBC, Boston, MA, USA, Version 1.4.1717©2009–2021, PBC). Die statistische Analyse für den Vergleich von Gruppenunterschieden bei normalverteilten Variablen erfolgte mittels *t*-Test. Nichtnormalverteilte Variablen wurden mittels Mann-Whitney-U- oder Kruskal-Wallis-Test verglichen. Eine univariate Regressionsanalyse wurde durchgeführt, um den Zusammenhang der Verwendung eines Fixateur externe und des Auftretens von Komplikationen im Vergleich zu Patienten ohne Fixateur externe zu analysieren. Als Signifikanzniveau wurde *p* < 0,05 festgelegt.

## Ergebnisse

### Patientenkollektiv

**Abb. 1:**
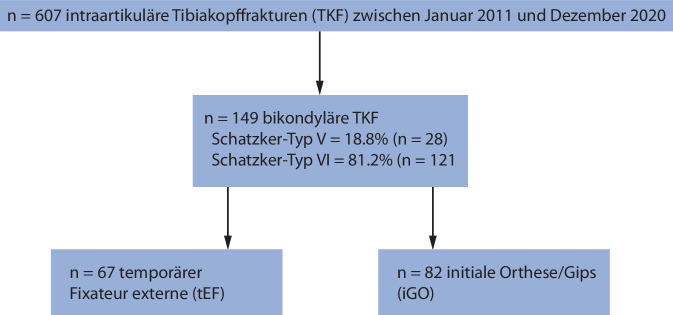
Flussdiagramm der Patientenselektion

Insgesamt konnten *n* = 607 intraartikuläre Tibiakopffrakturen (TKF) in dem Zeitraum vom Januar 2011 bis Dezember 2020 über das klinikinterne Tibiakopfregister identifiziert werden. Nach Anwendung der Ein- und Ausschlusskriterien konnten *n* = 149 bikondyläre TKF eingeschlossen werden. Davon wurden 28 Frakturen gemäß der Schatzker-Klassifikation als Typ V (18,8 %) und 121 Frakturen als Typ VI (81,2 %) klassifiziert. Von den 149 Frakturen wurden initial 67 mittels tEF und 82 mittels immobilisierenden Gipses/immobilisierender Orthese (iGO) behandelt. In der Gruppe, die initial mit einer Orthese oder einem Gips behandelt wurden, erfolgte in *n* = 0 Fällen ein Verfahrenswechsel zur Anlage eines Fixateur externe (Abb. 1).

Die tEF-Gruppe verzeichnet signifikant mehr männliche Patienten (58,2 %; *p* = 0,005), die einem jüngeren Patientenkollektiv zuzuordnen sind (49,9 ± 13 Jahre). Die iGO-Gruppe besteht aus signifikant mehr Frauen (61 %; *p* ≤ 0,001), die ca. 10 Jahre älter sind (57,7 ± 16 Jahre). Der American Society of Anesthesiologists (ASA) Score ist signifikant höher in der tEF-Gruppe (2,5 ± 0,8 vs. 2,1 ± 0,8; *p* = 0,014).

Als Hauptunfallursachen finden sich in der tEF-Gruppe „High-energy“-Traumata (Motorrad‑, Verkehrsunfall) mit 44,8 %. In der iGO-Gruppe sind überwiegend „Low-energy“-Traumata zu finden (Stolperstürze: 37,8 %).

Die Indikation zur tEF-Anlage erfolgte in 27,5 % der Fälle aufgrund eines polytraumatisierten Patienten, in 21,3 % aufgrund eines Kompartmentsyndroms, in 20 % aufgrund einer offenen Fraktur (Grade II und III nach Gustilo-Anderson [12]) und in 3,8 % aufgrund einer Luxationsstellung. In weiteren 27,5 % der Fälle wurde die Indikation zur tEF-Anlage aufgrund der Frakturmorphologie (ohne Luxationsstellung) gestellt.

Einen detaillierten Überblick über das Patientenkollektiv bietet Tab. 1.

**Tab. 1:** Detaillierte Informationen der Studienpopulation

Kriterien	Temporärer Fixateur externe	Primäre Osteosynthese	*p*-Wert
Männlich	58,2 % (49,9 ± 13 Jahre)	39,0 % (51,1 ± 12 Jahre)	0,005
Weiblich	41,8 % (62,5 ± 14 Jahre)	61,0 % (57,7 ± 16 Jahre)	< 0,001
BMI (kg/m^2^)	28,3 ± 17	26,9 ± 6	0,542
ASA	2,5 ± 0,8	2,1 ± 0,8	0,014
Operationsdauer (definitive Versorgung)	208 ± 87 min	172 ± 76 min	0,015
Zeit bis zur definitiven Osteosynthese	11,9 ± 12,4 Tage	4,3 ± 3,5 Tage	0,014
Unfallursache			
– Skilaufen	9 %	8,5 %	–
– Fahrradfahren	14,9 %	18,3 %	–
– Stolpersturz	19,4 %	37,8 %	–
– Sturz aus großer Höhe (> 3 m)	7,5 %	14,6 %	–
– Verkehrsunfall	16,4 %	3,7 %	–
– Motorradunfall	28,4 %	6,1 %	–
– Andere	4,5 %	11 %	–

### Zeit bis zur definitiven Osteosynthese

Die Zeit bis zur definitiven osteosynthetischen Versorgung ist in der tEF-Gruppe signifikant länger (*p* < 0,01; EF: 11,9 ± 12,4 Tage; iGO: 4,3 ± 3,5 Tage).

### Komplikationen

**Abb. 2:**
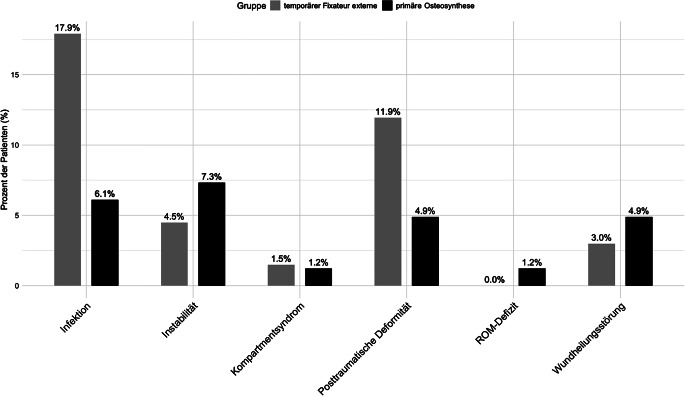
Übersicht Gesamtkomplikationen

Insgesamt konnten in dem Gesamtkollektiv *n* = 45 operationsbedürftige Komplikationen detektiert werden (Gesamtkollektiv: 30,2 %; EF: 40,3 % (*n* = 27); iGO: 22,0 % (*n* = 18)). Bei der tEF-Gruppe waren die häufigsten Revisionsindikationen Infektionen und posttraumatische Deformitäten, während in der iGO-Gruppe insbesondere Instabilitäten und Wundheilungsstörungen auftraten. Eine Übersicht der Komplikationen ist in Abb. 2 dargestellt.

### Regressionsanalyse

Die Regressionsanalyse zeigt keine signifikant erhöhte Gesamtkomplikationsrate für die tEF-Gruppe (Odds Ratio (OR) 1,97, 95 %-KI 0,90–4,37, *p* = 0,069).

**Abb. 3:**
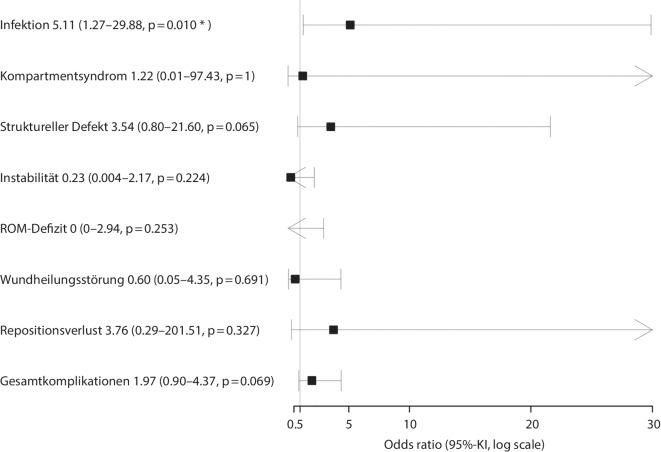
Komplikationen, assoziiert mit einem tEF

**Abb. 4:**
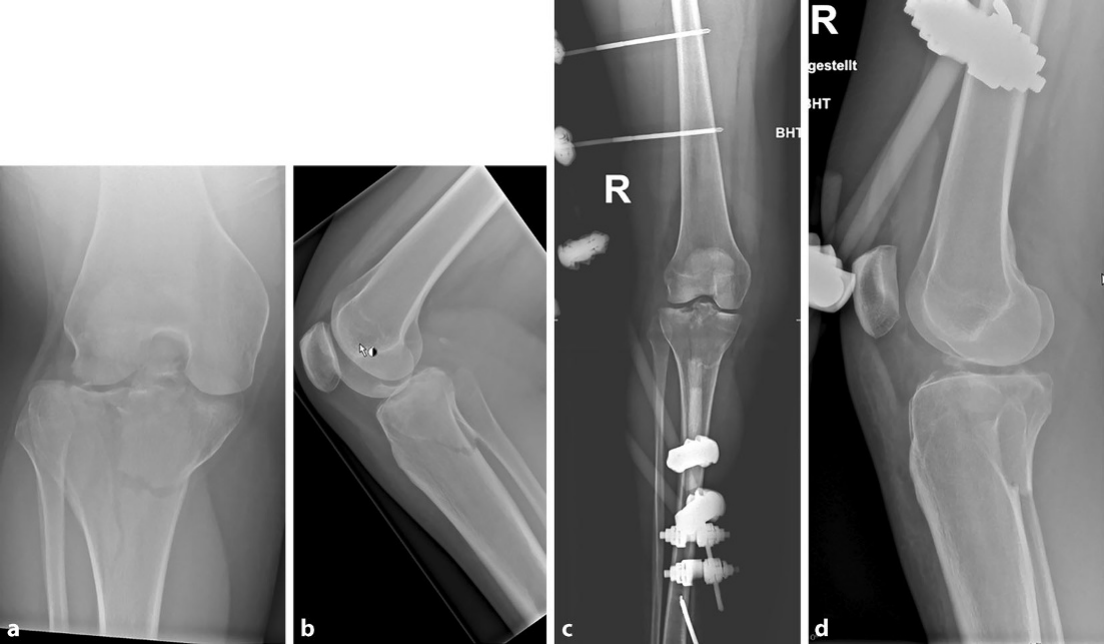
Aufnahmen einer 23-jährigen Patientin mit einer Tibiakopfluxationsfraktur, die initial mittels eines Fixateur externe versorgt wurde. **a**, **b** Röntgen anterior-posterior und lateral vor Fixateur-Anlage, **c**, **d** Röntgen anterior und lateral nach Fixateur-Anlage

Im Detail zeigen sich keine signifikanten Unterschiede bei Kompartmentsyndrom (OR 1,22, 95 %-KI 0,01–97,43, *p* = 1), Wundheilungsstörungen (OR 0,60, 95 %-KI 0,05–4,35, *p* = 0,691), strukturellen Defekten (OR 3,54, 95 %-KI 0,80–21,60, *p* = 0,065), ROM-Defizit (OR 0, 95 %-KI 0–2,94, *p* = 0,253) und postoperativer Kniegelenkinstabilität (OR 0,23, 95 %-KI 0,004–2,17, *p* = 0,224). Postoperative Infektionen zeigten eine signifikante Assoziation mit der Verwendung von tEF (OR 5,11, 95 %-KI 1,27–29,88, *p* = 0,01). Die Ergebnisse sind in Abb. 3 dargestellt; Fallbeispiele: Abb. 4 und 5.

**Abb. 5:**
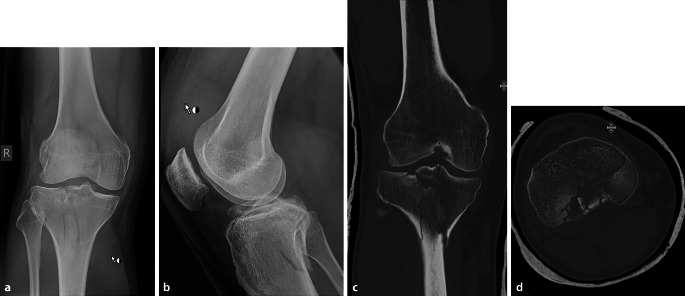
Aufnahmen eines 31-jährigen Patienten mit bikondylärer Tibiakopffraktur. Die initiale Versorgung erfolgte mittels Mecron-Schiene. Im Verlauf erfolgte dann die osteosynthetische Versorgung. **a**, **b** Röntgenbild anterior-posterior und lateral initial, **c**, **d** CT-Ausschnitt sagittal und axial nach Ruhigstellung in dorsaler Gipsschiene

## Diskussion

Die wichtigste Erkenntnis dieser retrospektiven Studie ist, dass die Gesamtkomplikationsrate von Patienten mit intraartikulären bikondylären TKF nicht abhängig von der initialen Versorgung mittels tEF oder ruhigstellender Orthese/ruhigstellendem Gips ist.

Kritisch zu diskutieren ist die signifikant erhöhte Zahl an postinterventionellen Infektionen nach tEF-Anlage. Hierbei ist es essenziell, Einflussfaktoren zu berücksichtigen, die einerseits klare Indikationen für die tEF-Anlage bei Tibiakopffrakturen darstellen, andererseits jedoch auch gleichzeitig das allgemeine postinterventionelle Infektionsrisiko erhöhen können (Tab. 2).

**Tab. 2:** Indikationen für die-Anlage eines temporären Fixateur externe

Harte Indikationen	Weiche Indikationen
Kritische Weichteilverhältnisse Offene Frakturen Polytraumatisierte Patienten Kompartmentsyndrom Luxationsstellung mit/ohne Gefäß‑/Nervenschaden	Komplexe Frakturmorphologie Initiale Luxation mit erfolgter Reposition

Hierzu zählen insbesondere kritische Weichteilverhältnisse, offene Frakturen sowie polytraumatisierte Patienten [3]. In diesem Kontext ist der Zusammenhang zwischen initial bestehenden kritischen Weichteilverhältnissen und der postinterventionellen Infektionsrate von besonderer Bedeutung [22]. Da kritische Weichteilverhältnisse und offene Frakturen harte Indikationen zur tEF-Anlage darstellen, muss in diesen Fällen mit einer erhöhten Infektionsrate gerechnet werden.

Im Gegensatz dazu zeigt ein isoliertes Hochrasanztrauma laut Laible et al. keinen signifikanten Einfluss auf die Infektionsrate [16]. Bei polytraumatisierten Patienten hingegen besteht eine signifikant erhöhte Infektionsanfälligkeit mit Infektionsraten von 60–90 %, was bei der Interpretation der Ergebnisse und der Planung zu berücksichtigen ist [21, 23, 26].

Zusätzlich zu diesen allgemeinen Infektionsrisiken scheint auch die tEF-Anlage einen alleinigen Risikofaktor für eine Infektion im Operationsgebiet darzustellen. Mahan et al. konnten in 74,8 % der Fälle nach Entfernung eines tEF positive mikrobiologische Kulturen an den Spitzen der Schrauben nachweisen [17, 18]. Im Gesamtkontext sollten die in der Literatur beschriebenen Infektionsraten von osteosynthetisch versorgten TPF bis zu 10 %, unabhängig von der initialen Versorgung, berücksichtigt werden [8, 22].

Die endgültige operative Versorgung erfolgte im Patientenkollektiv mit Orthese/Gips signifikant früher als im tEF-Kollektiv (*p* < 0,01; EF: 11,9 ± 12,4 Tage; iGO: 4,3 ± 3,5 Tage). Auch hier können die oben bereits genannten Faktoren „Weichteilverhältnisse“ und „polytraumatisierter Patient“ Einfluss auf die längere Zeitspanne in der tEF-Gruppe haben. Die Konsolidierung der Weichteile, die eine operative Versorgung ermöglicht, nimmt in beiden Gruppen einige Tage in Anspruch. Die Daten zeigen, dass eine Konsolidierung der Weichteile auch im Gips/in der Orthese möglich ist. In *n* = 0 Fällen erfolgte ein Verfahrenswechsel von der initialen Behandlung mittels Gipses oder Orthese zu der Anlage eines temporären Fixateur externe. Dies zeigt, dass bei komplexen Tibiakopffrakturen unter Gips- oder Orthesenbehandlung weder ein sekundärer Repositionsverlust noch eine zusätzliche Weichteilschädigung zu erwarten sind.

Die zentrale Frage dieser Studie ist die Frage nach der Indikation zur tEF-Anlage bei TKF. In der Literatur werden harte Indikationen zur tEF-Anlage benannt, welchen den polytraumatisierten Patienten, schwere Weichteilschäden bzw. offene Frakturen, Luxationsstellungen und manifeste Kompartmentsyndrome umfassen [3, 36].

Im betrachteten Patientenkollektiv lag in 70 % der Fälle eine oder mehrere dieser Indikationen vor. In 30 % der Fälle erfolgte die Indikationsstellung zur tEF-Anlage aufgrund der subjektiven Interpretation des Operateurs. Diese subjektiven Indikationen sollten vor dem Hintergrund der erhöhten Infektionsraten, des zunehmend alternden Patientenkollektivs mit Tibiakopffraktur und der knapper werdenden operativen Ressourcen kritisch hinterfragt werden [2, 11, 28].

Die hier vorliegende Studie zeigt, dass eine bikondyläre TKF ohne harte Indikation zur tEF-Anlage initial ohne Nachteil für den Patienten mit einem Gips/einer Orthese behandelt werden kann.

Die Ergebnisse der vorliegenden Studie sollten im Kontext ihrer Limitationen betrachtet werden. Die retrospektive Natur der Studie stellt einen Schwachpunkt der Studie dar. Eine prospektive, randomisierte Studie scheint jedoch aufgrund der oft komplexen Verletzungsmuster aus ethischen Gründen nicht durchführbar zu sein. Eine weitere Limitation besteht in der Heterogenität der Gruppen. Obwohl beide Gruppen ähnliche demografische Daten und Frakturmorphologien aufweisen, weist die tEF-Gruppe tendenziell ein schwereres Verletzungsmuster mit höherem Weichteilschaden auf. Weiterhin ist bezüglich der Gesamtkomplikationsrate ein Unterschied vorhanden, dieser erreicht jedoch keine statistische Signifikanz. Eine größere Fallzahl könnte möglicherweise zu einer signifikanten Differenz führen. Weiterhin können in dieser Studie keine Aussagen über das subjektive Outcome der Patienten sowie das mittel- und langfristige Outcome getroffen werden.

## Schlussfolgerung

Die Anwendung eines temporären Fixateur externe (tEF) vor der Osteosynthese bei komplexen Tibiakopffrakturen weist keine signifikant höheren Gesamtkomplikationsraten, aber höhere Infektionsraten im Vergleich zu einer primären Osteosynthese ohne tEF auf. Die Ergebnisse stützen die sichere Anwendung des tEF beim Vorliegen harter Indikationen. Fehlen diese harten Indikationen, kann die primäre Immobilisierung im Gips/in der Orthese erfolgen. Die individualisierte Entscheidung der tEF-Anlage unter Berücksichtigung der patientenspezifischen und klinikinternen Ressourcen bleibt wichtig.

## Fazit für die Praxis

- Die Gesamtkomplikationsrate bei TKF ist nicht von der initialen Immobilisierung mittels Fixateur externe oder Gips/Orthese abhängig.
- Beim Vorliegen harter Indikationen sollte eine Fixateur-Anlage notfallmäßig erfolgen.
- Beim Fehlen harter Indikationen für die Fixateur-Anlage kann eine primäre Immobilisierung mittels Gips/Orthese erfolgen.

## Data Availability

Die erhobenen Datensätze können auf begründete Anfrage in anonymisierter Form beim korrespondierenden Autor angefordert werden.
